# Motility Subpopulations with Distinct Motility Characteristics Using Swim-Up-Selected Sperm Cells from Norwegian Red Bulls: Effects of Freezing–Thawing and Between-Bull Variation

**DOI:** 10.3390/biology12081086

**Published:** 2023-08-03

**Authors:** Anne Hege Alm-Kristiansen

**Affiliations:** Faculty of Applied Ecology, Agricultural Sciences and Biotechnology, Inland Norway University of Applied Sciences, 2418 Hamar, Norway; anne.almkristiansen@inn.no

**Keywords:** bovine, sperm selection, cryopreservation, CASA

## Abstract

**Simple Summary:**

For the breeding of cattle using artificial insemination, the cryopreservation of sperm cells is an essential tool. To predict the male fertility potential, it is of utmost importance to control the sperm quality after freezing–thawing, before sending the semen samples to be used at farms for inseminations. Sperm motility is a crucial parameter to keep track of and can be analyzed using the Computer-Assisted Sperm Analyzer, which can accurately and objectively address the question of sperm function with a user-friendly method at the AI station. The sperm cells can be divided into different subpopulations according to swimming pattern and velocity, like rapid progressive, rapid non-progressive and slow-motility spermatozoa. In this study, Norwegian Red bull sperm motility was studied and compared between fresh and frozen–thawed sperm cells after more in vivo-like conditions, using sperm selection via a swim-up method and incubation at body temperature over time. The difference between the bulls was mainly due to the subpopulation with a rapid progressive swimming pattern, which was also the only population significantly correlating with fresh and frozen–thawed spermatozoa from the same bull. These results indicate that rapid, progressive sperm cells are the possible indicators of bull spermatozoa functionality and freezability.

**Abstract:**

Discrete subpopulations of motile sperm cells have been found for several species and are implicated to be important for sperm functionality. The aim of this present study was to examine the motile subpopulations in swim-up-selected bull spermatozoa and the relationship between subpopulations in fresh and frozen–thawed sperm cells. In experiment 1, swim-up (SWUP)-selected and non-selected (control) sperm cells were analyzed using a Computer-Assisted Sperm Analyzer (CASA). In experiment 2, the semen from nine bulls was cryopreserved and analyzed using CASA both before and after freezing and after incubation at physiological temperatures. The SWUP population had a higher proportion of total motility, progressivity, and velocity compared to the control (*p* < 0.05). Likewise, both incubation over time and cryopreservation affected motility and motility parameters (*p* < 0.05). The population of rapid progressive (RapidP) sperm cells dominated the SWUP fraction and was higher than in the control samples (*p* < 0.05). Furthermore, RapidP was also the main part of fresh semen, but decreased significantly over time during incubation and due to cryopreservation. In conclusion, RapidP was the main population in SWUP-selected spermatozoa and seems to be an important subpopulation contributing to the differences between treatments and in response to the freezing of sperm cells.

## 1. Introduction

The breeding of dairy cattle is mainly conducted via the artificial insemination of frozen–thawed semen. However, cryopreservation induces injuries in spermatozoa with loss in viability and motility, leading to impaired sperm functionality and reduced fertility [1]. Motility is measured at the AI station routinely as subjective motility using phase-contrast microscopy, or more preferably, using Computer-Assisted Sperm Analyzer (CASA). Sperm motility is important for sperm transport in the female reproductive tract and the penetration of zona pellucida [2]. Although motility is considered a compensable trait [3], CASA parameters have been found to be correlated to bull fertility [4,5,6,7,8]. The classical approach is a measure of total and progressive motility, and uses the mean values of the semen sample. However, the ejaculates of many mammalian species contain subpopulations of spermatozoa with various swimming properties. In this regard, CASA also gives the opportunity to study the diversity of sperm cell subpopulations in more detail concerning different motility patterns [9]. Differentiating sperm cells into distinct subpopulations is likely a more meaningful approach in correlating motility to fertility than the mean or median of the different kinematic parameters of the whole sperm cell population. Identifications of subpopulations have been performed for several species, including boar, donkey, red deer, and bull [10,11,12]. When analyzing the motility of spermatozoa, researchers have found typically three to four main subpopulations, even from quite diverse species [9]. There are some similarities between the following populations: one population consisting of spermatozoa swimming with low velocity, either progressive or non-progressive; next, a population with high velocity and non-progressive trajectories; and finally, a subpopulation with high velocity and high progressive motility. Overall, subpopulations recognized by different species reflect variations in swimming speed and progressivity. Those differences can be assumed to reflect discrepancies in sperm physiology and functionality. Therefore, identifying diverse sperm subpopulations with a superior capability to reach the female oviducts might be of the utmost importance to improve the accuracy of sperm quality assessments. To identify the sperm cells first approaching the fertilization site in vitro, it is desirable to use conditions as close as possible to the physical and physiological environments of the female reproductive tract [13]. The ions in the oviductal fluid support and modulate sperm motility [14]. Thus, using a combination of a medium containing ions that are important for sperm motility [15] and sperm selection, like swim-up, could be a possible model system [16,17]. Importantly, at the AI station, the sperm quality analysis must be performed quickly, accurately, and at a low cost. By combining knowledge from the research of different sperm subpopulations with user-friendly CASA systems available at most cattle AI stations, a more manageable analysis has the potential of being established. 

The aim of this present study was to examine the bull sperm motility subpopulations in Norwegian Red bulls by studying the following: (1) the sperm subpopulations in swim-up-selected spermatozoa, (2) the existence of the different sperm subpopulations in fresh ejaculates, and (3) the effects of freezing–thawing and post-thaw incubation on the distribution of spermatozoa within the different subpopulations.

## 2. Materials and Methods

### 2.1. Semen Processing and Freezing

Semen was collected from young Norwegian Red bulls during regular semen production at the Geno AI center (Geno Breeding and AI Association, Hamar, Norway). Two ejaculates per bull were collected within 15 min with an artificial vagina and pooled. Further processing of ejaculates was performed when motility was at least 70% and morphology above 85% normality. Semen was processed using the Biladyl^®^ extender (Minitube, Verona, WI, USA, 13500/0004-0006) in a two-step dilution procedure to a final concentration of 15 million spermatozoa per straw (French mini straws, IMV, L’Aigle, France) before cryopreservation, as described earlier [18].

### 2.2. Experimental Setup

For experiment 1, cryopreserved semen from four bulls were used for the swim-up experiments. The semen samples were thawed for 1 min at 37 °C, centrifugated for 5 min at 800× *g* and resolved in modified spTalp-H [15,19] (87 mM NaCl, 3.1 mM KCl, 0.4 mM MgCl_2_, 0.3 mM NaH_2_PO_4_, 2.0 mM CaCl_2_, 20 mM Hepes sodium salt, 20 mM Hepes acid, 10 mM NaHCO_3_, 1 mM sodium pyruvate, 5 mM glucose, 21.6 mM sodium lactate, 6 mg/mL BSA, 50 µg/mL gentamicin, pH 7.4, osmolarity of 292 mOsm) to 70 million spermatozoa/mL. For the swim-up procedure, two parallels of 100 µL semen sample were carefully pipetted to the bottom of two 15 mL tubes with 500 µL prewarmed spTalp-H and incubated for one hour at 38 °C to allow the sperm cells to swim up. Thereafter, 400 µL (the upper fraction) of each tube was carefully removed and the two parallels pooled in prewarmed Eppendorf tubes, before centrifugating for 10 min at 300× *g*. Next, 600 µL supernatant was removed before the pellets were carefully resolved in the remaining solution (swim-up fraction, SWUP for short hereafter). One aliquot was incubated for one hour at 38 °C as the control, and further diluted 1:3 in spTalp-H before the CASA analysis.

For experiment 2, the semen samples from nine bulls were analyzed using CASA for both fresh and cryopreserved samples. Part of each fresh semen ejaculate sample was diluted directly in spTalp-H to a concentration of 20 million sperm cells per mL and analyzed after incubation at 38 °C for 5 min (T0), three (T3) and six (T6) hours. The cryopreserved semen samples from the same bulls were analyzed frozen–thawed (FT; T0) and after three hours (T3) at 38 °C, both in spTalp-H.

### 2.3. Computer-Assisted Sperm Analysis (CASA)

Sperm motility parameters were assessed using the CASA system (Sperm Class Analyzer^®^ version 6.1, Microptic SL, Barcelona, Spain). The semen samples were incubated for 5 min at 38 °C, rotated 180° five times and 3 µL was put on a Leja^®^ 4 chamber slide (Nieuw-Vennep, The Netherlands). Instrument settings were 50 frames per second and 30 images per sample. Sperm cells were identified by the sperm head area of 30–70 µm^2^. Eight fields were collected per parallel. The following kinematic parameters were recorded: velocity average path (VAP, µm/s), velocity curved line (VCL, µm/s), velocity straight line (VSL, µm/s), straightness (STR) of the average path defined as the VSL/VAP (%) ratio, linearity (LIN) of the curvilinear path defined as the VSL/VCL (%) ratio, Wobble (WOB) defined as the VAP/VCL (%) ratio, amplitude of the lateral head displacement (ALH, µm), and beat cross-frequency (BCF, Hz). Total motility (MOT) was defined as sperm cells with a VCL ˃ 15 µm/s, and progressive motility (PROG) was defined as sperm cells with an STR ˃ 70. In addition, the use of threshold settings in SCA 6.1 allow for the classification of spermatozoa into subpopulations. The playback function was used to visually control the limits according to the visual motility trajectories. The subgroups were defined as following; RapidP; VCL > 50 µm/s and STR > 70, RapidNP VCL > 50 µm/s and STR < 70 and Slow motile; VCL< 50 µm/s. Single-cell analysis was performed through the extraction of data from all analyzed samples at T0, resulting in single-cell data from 5338 SWUP spermatozoa.

### 2.4. Statistical Analyses

The data from spermatozoa obtained in the SWUP selection experiments and fresh semen experiment from T0 was extracted as single-cell data, where each sperm cell was represented by kinematic parameters and categorized into subpopulations, as described above. The data are represented as the mean, with standard deviation (SD) and 95% confidence intervals. An analysis of variance combined with a Tukey test was performed for multiple pairwise comparisons of the parameter means. The relationship for subpopulations between fresh and FT semen was determined by Pearson correlation coefficients. The statistical analysis was performed using R, version 3.1.2 (http://www.r-project.org (accessed on 29 March 2019)), and the differences were considered significant when *p* < 0.05.

## 3. Results

### 3.1. Changes in the Mean Motility Parameters after Selection of Frozen–Thawed Spermatozoa by Swim-Up

**Figure 1:**
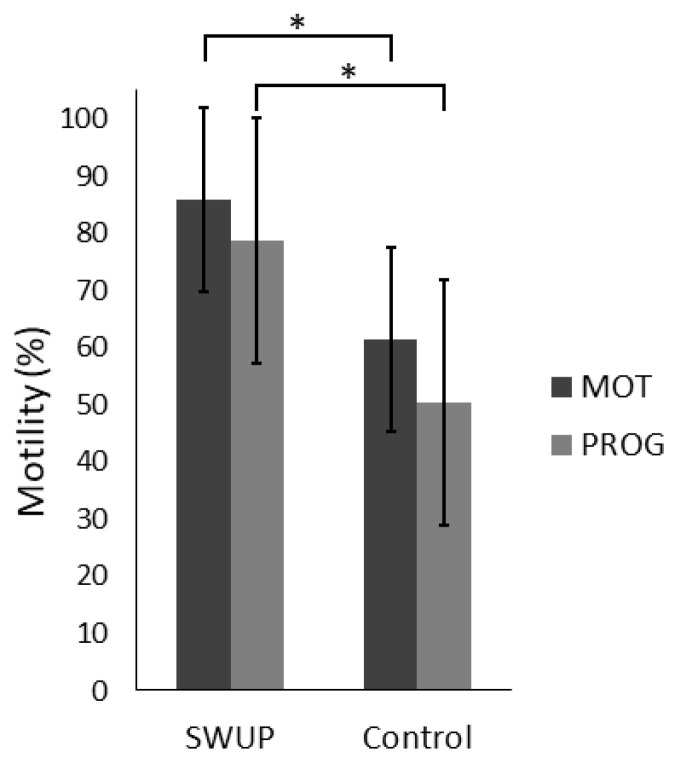
Bull sperm cells were selected by swim-up (SWUP) for one hour at 38 °C or incubated in spTalp-H for the same period as the control at 38 °C and analyzed using CASA. The experiment was repeated four times with frozen–thawed semen from four different bulls. Results are shown as the percentage mean total motility (MOT) and progressive motility (PROG) with a 95% confidence interval. * Mean values within parameter differ significantly (*p* < 0.05).

**Figure 2:**
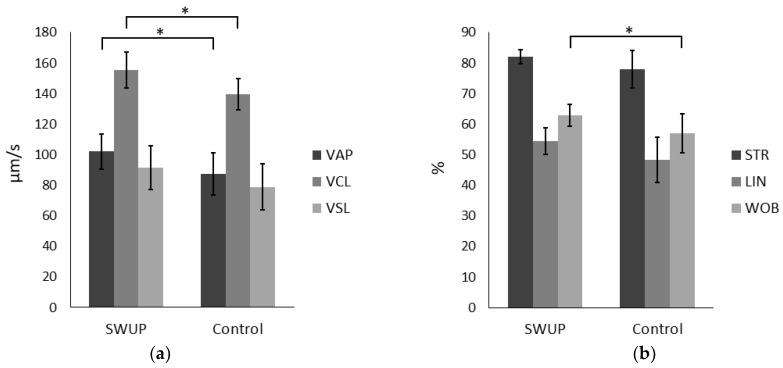

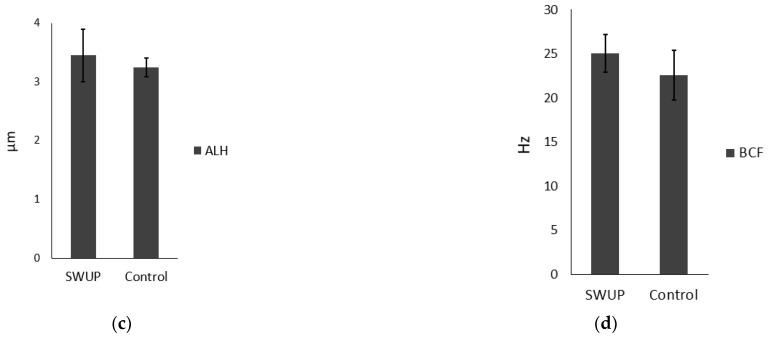
Bull sperm cells were selected by swim-up (SWUP) or incubated in spTalp-H for the same period as the control and analyzed using CASA. The experiment was repeated four times with frozen–thawed semen from four different bulls. Kinematic parameters: (**a**) velocity average path (VAP, µm/s), velocity curved line (VCL, µm/s), velocity straight line (VSL, µm/s), (**b**) straightness (STR) defined as the VSL/VAP (%) ratio, linearity (LIN) defined as the VSL/VCL (%) ratio, Wobble (WOB) defined as the VAP/VCL (%) ratio, (**c**) amplitude of lateral head displacement (ALH, µm) and (**d**) beat cross-frequency (BCF, Hz). Results are shown as the mean kinematic parameter with 95 % confidence interval. * Mean values between parameters differ significantly (*p* < 0.05).

The selection of sperm cells by swim-up (SWUP) significantly improved the proportion of motile sperm cells (Figure 1). Likewise, the velocity parameters VAP and VCL increased for SWUP spermatozoa compared to the control (Figure 2, *p* < 0.05). The parameters STR, LIN and WOB were all higher for the SWUP-selected population, indicating a more linear swimming pattern, though, only WOB was significantly changed (*p* < 0.05). There were no significant differences in the ALH (*p* > 0.05) or BCF (*p* > 0.05). 

### 3.2. Characterization of Motile Sperm Subpopulations Selected by Swim-Up

**Figure 3:**
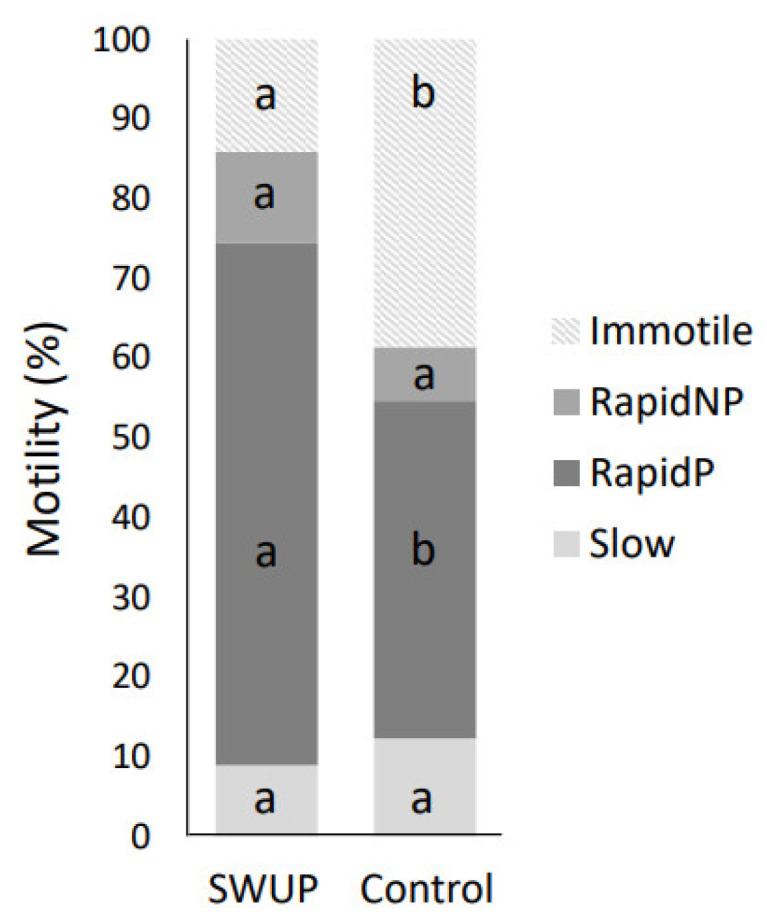
Distribution of sperm cells into motility subpopulations: immotile, rapid non-progressive (RapidNP), rapid progressive (RapidP) and Slow motility, of sperm cells selected by swim-up (SWUP) or incubated for one hour as the control (*n* = 4) and analyzed using the Computer-Assisted Sperm Analyzer (CASA). Values within the subpopulation with distinct letters differ significantly (*p* < 0.05).

**Figure 4:**
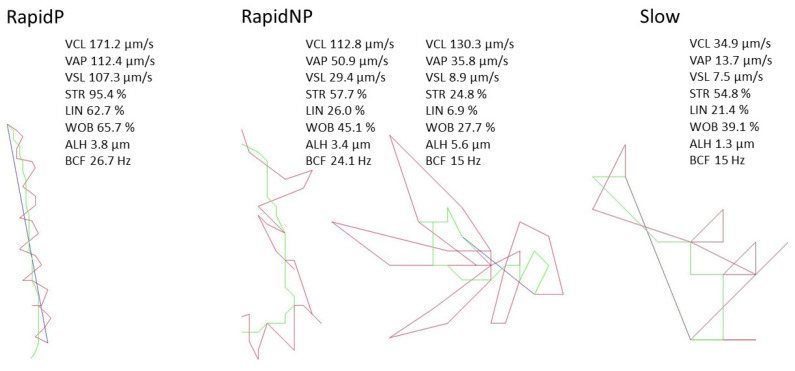
Representative trajectories of bull sperm cells within different motility subpopulations: rapid progressive (RapidP), rapid non-progressive (RapidNP) and Slow motility (Slow), with the kinematic parameters for the individual spermatozoa shown. Velocity average path (VAP, µm/s), velocity curved line (VCL, µm/s), velocity straight line (VSL, µm/s), straightness (STR), linearity (LIN), Wobble (WOB), amplitude of lateral head displacement (ALH, µm) and beat cross frequency (BCF, Hz). Bull sperm cells were analyzed by the Sperm Class Analyzer^®^ (version 6.1, Microptic SL, Barcelona, Spain)and divided into different subpopulations according to velocity and straightness. Red lines; VCL, green lines; VAP, and blue lines; VSL.

The distribution of the different motility subpopulations for the SWUP-selected spermatozoa is shown in Figure 3, and pictures of the sperm cell-corresponding movement are shown in Figure 4. The summary statistics for the kinematic parameters of the different subpopulations are given in Table 1 and Table 2. Rapid non-progressive (RapidNP) sperm cells have a high VCL and low straightness and linearity. The subpopulation defined as Slow include sperm cells of which the movements are characterized by a low VCL, VSL, and VAP. The third subpopulation swims with a rapid progressive (RapidP) swimming pattern, having a high VCL, VAP and VSL, and high STR and LIN. ALH was higher in RapidNP than RapidP (*p* < 0.05 for SWUP, *p* > 0.05 for control), but was lowest for the Slow sperm cell population (*p* < 0.05) in both the SWUP-selected sperm population and for the control sample (*p* < 0.05). BCF though, was higher in RapidP than in RapidNP, which, again, was higher than the Slow population (*p* < 0.05). 

The RapidP population was found to be the subpopulation increasing most after the swim-up selection procedure compared to the control spermatozoa (Figure 3, *p* < 0.05), while the percentage of RapidNP and Slow motile spermatozoa did not differ significantly.

### 3.3. Changes in Motile Sperm Kinematic Parameters after Incubation and Post-Freezing–Thawing

**Figure 5:**
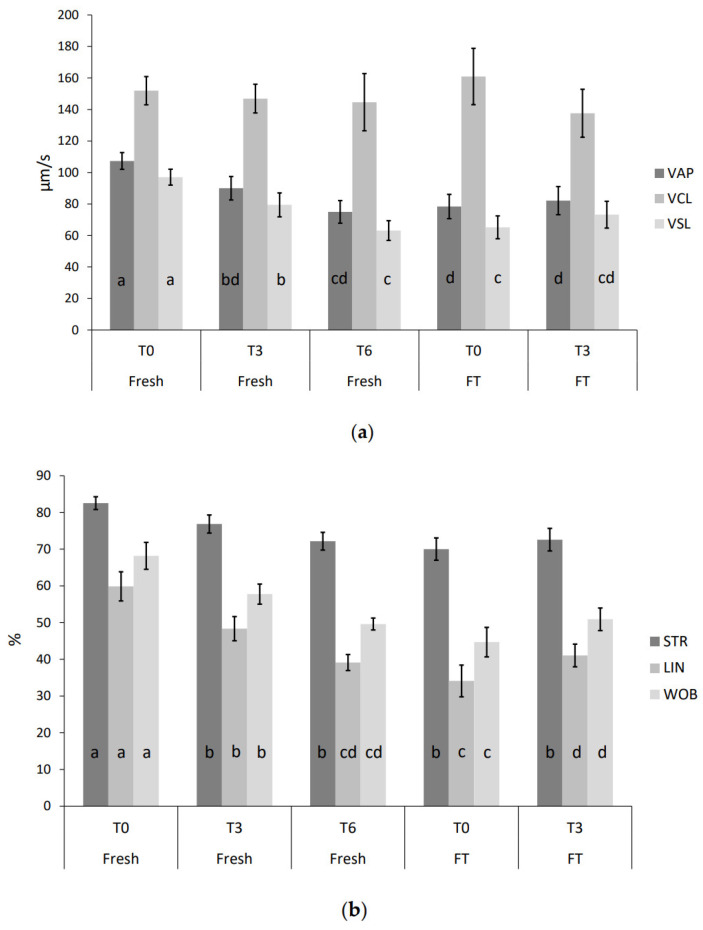

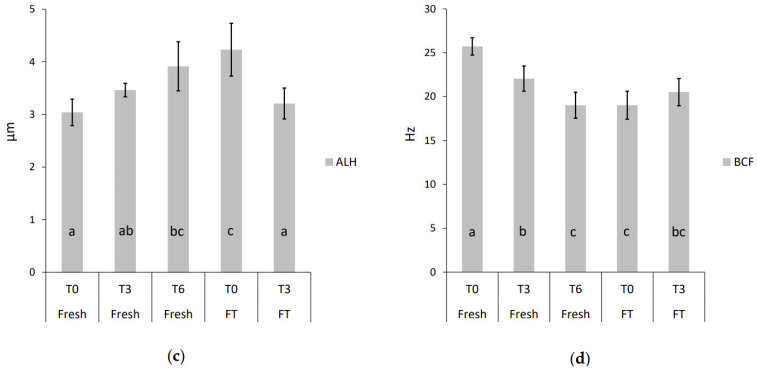
Fresh semen from NFR bull was incubated in spTalp-H post-collection (T0) and for three (T3) and six hours (T6) at 38 °C. Frozen–thawed (FT) semen was incubated post-thaw and analyzed at T0 and T3. Semen samples was analyzed using CASA and presented as the mean (±95% confidence interval). The experiment was performed with semen from nine different bulls. Kinematic parameters: (**a**) velocity average path (VAP, µm/s), velocity curved line (VCL, µm/s), velocity straight line (VSL, µm/s), (**b**) straightness (STR), linearity (LIN), Wobble (WOB), (**c**) amplitude of lateral head displacement (ALH, µm) and (**d**) beat cross frequency (BCF, Hz). Parameters with distinct letters differ significantly (*p* < 0.05).

As expected, the frozen–thawed (FT) semen decreased total motility and progressive motility as compared to fresh sperm cells (*p* < 0.05). Furthermore, cryopreservation induced changes in kinematic parameters (Figure 5). Both VAP and VSL decreased for FT sperm cells (*p* < 0.05), but not VCL (*p* > 0.05). The linearity of FT spermatozoa decreased (*p* < 0.05), while ALH increased for FT at T0 compared to the fresh semen at T0 (*p* < 0.05). Interestingly, velocity parameters for the fresh semen after incubation for three (T3) and six hours (T6) were comparable to the parameters for FT at T0. Moreover, the swimming pattern of the fresh sperm cells also changed during incubation. STR, LIN, WOB and BCF all decreased, while ALH increased. Furthermore, the kinematic parameters for FT semen also changed at T3, as VCL was slightly decreased (*p* > 0.05), and the linearity was increased (*p* < 0.05).

### 3.4. Characterization of Motile Sperm Subpopulations in Fresh Semen

**Figure 6:**
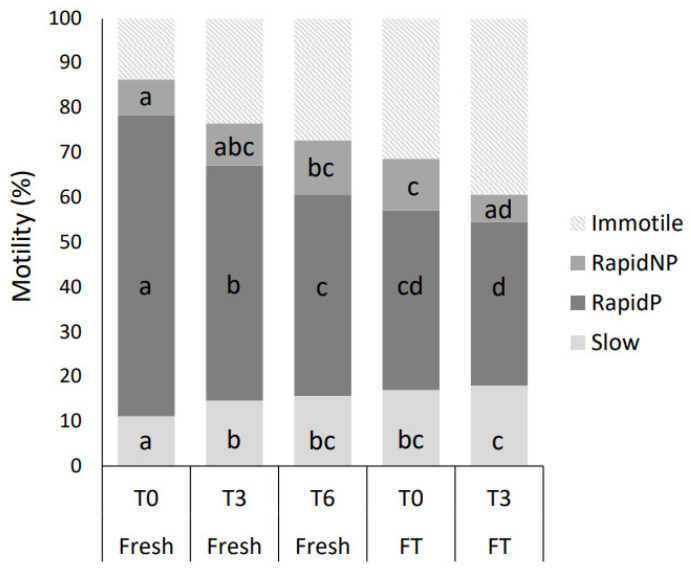
Fresh semen from nine bulls was incubated in spTalp-H post-collection (T0), for three (T3) and six hours (T6) at 38 °C. Frozen–thawed (FT) semen was incubated post-thaw (T0) and for three hours (T3). Semen samples were analyzed using CASA. Distribution of sperm cells into motility subpopulations: immotile, rapid non-progressive (RapidNP), rapid progressive (RapidP) and Slow motile. Values within subpopulation with unique letters are significantly different (*p* < 0.05).

Kinematic parameters for the different subpopulations in fresh semen are shown in Table 3. The total motility of the fresh semen was 86.3% at T0, where the main population was RapidP (67.2%), which also decreased the most during incubation at body temperature (*p* < 0.05, Figure 6).

### 3.5. Changes in Motile Sperm Subpopulations after Incubation and Post-Freezing–Thawing

The proportion of spermatozoa allocated to the different subpopulations changed significantly during incubation and after cryopreservation for all subpopulations (Figure 6, *p* < 0.05). The subpopulation RapidP was higher in fresh semen compared to FT semen. However, during incubation over time at body temperature, RapidP decreased. Furthermore, at T6, the fresh semen was not significantly different from FT at T0 (*p* >0.05); however, there was a significant difference from FT at T3. Likewise, there was no significant difference in the Slow subpopulation between the fresh semen at T6 and FT at T0 or T3; however, the proportion of the Slow spermatozoa increased for the fresh spermatozoa incubated over time post-collection (*p* < 0.05). RapidNP, on the other hand, slightly increased over time for the fresh semen, and increased due to freezing–thawing compared to fresh semen at T0 (*p* < 0.05). However, in contrast, RapidNP decreased again over time for FT at T3 compared to FT at T0 (*p* < 0.05). The Slow population did not significantly vary during post-thaw incubation. 

Distribution of motile subpopulations for individual bulls at T0 for fresh and FT sperm cells is shown in Supplementary Figure S1. Furthermore, it was observed that the proportion of RapidP spermatozoa decreased for all bulls after cryopreservation (*p* < 0.05). Interestingly, correlation analysis showed that for the different subpopulations, only RapidP was significantly correlated between the fresh and frozen semen at T0 (r = 0.681, *p* < 0.05).

## 4. Discussion

The differences in subpopulation swimming patterns can be assumed to reflect differences in functional competence, and by examining the bull sperm motility subpopulations of SWUP-selected spermatozoa, the sperm cells first approaching the fertilization site were mimicked in vitro. The SWUP-selected proportion of sperm cells in this study consisted mainly of rapid progressive (RapidP) sperm cells. Likewise, the RapidP spermatozoa also dominated fresh semen, and correlated between bulls for the fresh and frozen semen samples. Taken together, these results imply that the RapidP population describes an important functionality of sperm cells. The sperm cells swimming up may be a representative in vitro measure of the motile sperm cells present in the reproductive tract after insemination, contributing to a successful fertilization. 

As expected, freezing–thawing of spermatozoa led to reduced post-thaw motility, being evident as differences in allocation of spermatozoa to different subpopulations. It is well known that cryopreservation reduces fertility (reviewed in [1]), and examination of variations in motility subpopulations can provide increased knowledge of sperm functionality. In this study, RapidP was the subpopulation changing the most in response to cryopreservation. These results are in line with earlier results from other bovine breeds [11,20]. Furthermore, we found the population of RapidP to be correlated between the fresh and frozen–thawed semen from the same ejaculates, which supports the hypothesis of a functional sperm cell population interesting to consider as a sperm quality parameter of cryopreserved spermatozoa.

The fast-linear moving sperm population has been suggested as an indicator of fertility in different species [11,20,21,22,23,24]. It is interesting to note that the loss of motility during cooling and freezing in this study mostly decreases in the RapidP population, in line with other results [11]. Moreover, there was a marked reduction in RapidP over the six-hour incubation for the fresh semen. Thus, for FT semen, there was a decrease in the percentage of rapid spermatozoa, however, mainly within the non-progressive population. 

Single-cell data for the different treatment of spermatozoa (fresh, frozen–thawed, SWUP) showed similar kinematic parameter characteristics of sperm cells within the different subpopulations. However, the proportion of spermatozoa allocating to the different subpopulations differ as a result of treatment. This is also supported by differences in kinematic parameters owing to sperm handling. The fresh sperm cells swam with a rather linear pattern at T0; however, over time, linearity decreased. It was also observed that the linearity of FT spermatozoa was decreased compared to the fresh semen. However, the pattern of FT semen was more alike the pattern of fresh sperm cells incubated over time at a physiological temperature. This could be caused by changes in motility complying to capacitation, as it is well known that cryopreservation leads to changes in sperm cells that resembles capacitation [25,26]. Moreover, one could speculate whether fresh sperm cells might have the same changes due to stress induced during incubation at body temperature. Given that capacitation is tightly coupled to the level of reactive oxygen species (ROS) in spermatozoa [27,28,29] and the observations of motility decrease over time due to ROS increase in other studies [30,31,32], this could be a plausible explanation of the motility reduction over time in this study. Moreover, the observation of the increased percentage of hyperactive sperm cells for fresh sperm cells over time (Supplementary Figure S2), combined with the fact that hyperactivity increases with capacitation [28], supports the above hypothesis of stress-induced capacitation in fresh sperm. Further studies of the functional properties of the different sperm subpopulations, and specifically the RapidP population, will be interesting to follow. Gallo et al. reported that mitochondrial membrane potential is correlated with motility for bovine spermatozoa [33]. High mitochondrial membrane potential was also concurrent with high motility in swim-up-selected spermatozoa compared with control cells [34]. It is well known that sperm motility depends on energy supply [35], and a higher ATP level is expected for a semen sample with higher motility, since ATP is needed for swimming properties, and the ATP level and motility in semen samples are correlated [36].

Swim-up selection of sperm cells has been associated with fertility in earlier studies, since the concentration of motile sperm cells after swim-up was found to be correlated with a nonreturn rate in cattle [16,17]. Moreover, semen samples from bulls with higher fertility had a higher viability after SWUP selection than bulls with lower fertility [37]. In this study, the SWUP population predominantly consisted of RapidP spermatozoa, indicating RapidP to be the spermatozoa with the highest probability of resulting in a successful fertilization. Interestingly, a relationship between the sperm subpopulation with rapid progressive swimming pattern and in vitro fertility has been found for frozen–thawed spermatozoa from Holstein bulls [38] and dogs [23]. The importance of fast-moving sperm cells for fertility was also supported by Hidalgo et al. (2021); however, this study reported the rapid non-progressive sperm cells to be correlated with the fertility outcome [39]. 

For studies of motile subpopulations, it is important to keep in mind the importance of in vivo female reproductive tract conditions [13]. Another method for the selection of sperm cells mimicking travel to the fertilization site is a microfluidic device [40,41], reviewed in [13], which has been found to produce related results to the swim-up method [42]. Thus, for use at the AI centers, the swim-up procedure takes at least one hour to perform. Likewise, some extra time using microfluidics must be taken into account, so it is preferred to use these methods to achieve more information about sperm function to thereafter design a simple and effective method for the evaluation of sperm quality using CASA analysis of the extended semen directly, using specific CASA settings for different subpopulations. More knowledge of the different motile subpopulation contributions to increased fertility is valuable for both the breeding industry and in research. 

## 5. Conclusions

The present study showed that RapidP was the main subpopulation of both SWUP-selected spermatozoa and fresh sperm cells. Furthermore, the proportion of RapidP correlated between the fresh and frozen semen samples, providing information about the individual bull semen freezability and response to different treatments. The frequency distribution of spermatozoa to different subpopulations most likely represents sperm cells in different physiological states, manifested by their different swimming pattern. Future studies are needed to examine the relevance of different subpopulations for the functionality and fertilizing capacity of spermatozoa. The assessment of sperm cell quality is important in semen preservation, and the results of research will contribute to the standardization of protocols and further knowledge for reliable limits for production dose approval at AI stations. 

## Figures and Tables

**Table 1:** Kinematic parameters for motility subpopulations for swim–up-selected (SWUP) sperm cells. A Sperm Class Analyzer was used to divide the sperm populations into rapid non-progressive (RapidNP), rapid progressive (RapidP), and Slow motile sperm cells. The results are given as the mean, SD, and 95% confidence intervals (CI; confidence interval, U; upper, L; lower).

Parameter		RapidNP	RapidP	Slow
VCL (µm/s)	Mean	140.3 ^a^	174.6 ^b^	30.0 ^c^
	SD	56.5	42.4	10.4
	CI-L	136.0	173.2	29.0
	CI-U	145.0	175.9	30.9
VSL (µm/s)	Mean	34.0 ^a^	113.1 ^b^	6.36 ^c^
	SD	23.4	33.5	8.29
	CI-L	32.2	112.0	5.61
	CI-U	35.8	114.2	7.11
VAP (µm/s)	Mean	71.0 ^a^	120.7 ^b^	12.0 ^c^
	SD	39.2	31.9	8.81
	CI-L	68.0	119.6	11.2
	CI-U	73.9	121.7	12.8
LIN (%)	Mean	22.9 ^a^	64.9 ^b^	19.8 ^c^
	SD	12.5	12.9	21.3
	CI-L	21.9	64.5	17.8
	CI-U	23.8	65.3	21.7
STR (%)	Mean	45.7 ^a^	92.9b ^b^	45.1 ^a^
	SD	17.9	6.7	32.9
	CI-L	44.3	92.7	42.1
	CI-U	47.0	93.1	48.1
WOB (%)	Mean	48.0 ^a^	69.5 ^b^	38.6 ^c^
	SD	15.7	11.0	22.0
	CI-L	46.8	69.1	36.6
	CI-U	49.2	69.8	40.6
ALH (µm)	Mean	4.26 ^a^	3.58 ^b^	1.32 ^c^
	SD	1.60	1.12	0.41
	CI-L	4.14	3.55	1.29
	CI-U	4.38	3.62	1.36
BCF (Hz)	Mean	19.9 ^a^	28.0 ^b^	8.90 ^c^
	SD	6.52	7.16	7.56
	CI-L	19.4	27.8	8.21
	CI-U	20.4	28.2	9.59

^a–c^ Values in rows with distinct letters differ significantly (*p* < 0.05).

**Table 2:** Kinematic parameters for motility subpopulations for frozen–thawed control sperm cells. Spermatozoa post freezing–thawing were incubated in spTALP-H for one hour before analysis. A Sperm Class Analyzer was used to divide the sperm populations into rapid non-progressive (RapidNP), rapid progressive (RapidP), and Slow motile sperm cells. The results are given as the mean, SD, and 95% confidence interval (CI; confidence interval, U; upper, L; lower).

Parameter		RapidNP	RapidP	Slow
VCL (µm/s)	Mean	121.1 ^a^	175.2 ^b^	26.4 ^c^
	SD	65.3	38.6	10.2
	CI-L	110.3	172.8	25.2
	CI-U	131.9	177.6	27.6
VSL (µm/s)	Mean	26.7 ^a^	110.3 ^b^	4.94 ^c^
	SD	23.3	29.1	7.19
	CI-L	22.9	108.5	4.08
	CI-U	30.6	112.1	5.80
VAP (µm/s)	Mean	53.4 ^a^	117.1 ^b^	9.57 ^c^
	SD	36.9	27.1	8.20
	CI-L	47.3	115.5	8.59
	CI-U	59.5	118.8	10.6
LIN (%)	Mean	20.0 ^a^	63.1 ^b^	17.6 ^a^
	SD	12.7	12.3	20.1
	CI-L	17.9	62.3	15.2
	CI-U	22.0	63.8	20.0
STR (%)	Mean	45.2 ^a^	93.2 ^b^	44.3 ^a^
	SD	20.0	6.6	33.6
	CI-L	41.9	92.8	40.3
	CI-U	48.5	93.6	48.4
WOB (%)	Mean	41.8 ^a^	67.3 ^b^	34.8 ^c^
	SD	16.6	10.5	22.3
	CI-L	39.1	66.6	32.1
	CI-U	44.6	67.9	37.5
ALH (µm)	Mean	3.83 ^a^	3.67 ^a^	1.24 ^b^
	SD	1.92	1.04	0.43
	CI-L	3.52	3.61	1.19
	CI-U	4.15	3.74	1.29
BCF (Hz)	Mean	18.1 ^a^	28.4 ^b^	7.16 ^c^
	SD	7.18	6.49	6.03
	CI-L	16.9	28.0	6.44
	CI-U	19.3	28.8	7.88

^a–c^ Values in rows with distinct letters differ significantly (*p* < 0.05).

**Table 3:** Kinematic parameters for motility subpopulations for fresh bull sperm cells. Spermatozoa were incubated in spTALP-H before analysis. A Sperm Class Analyzer was used to divide the sperm population into rapid non-progressive (RapidNP), rapid progressive (RapidP), and Slow motile sperm cells. The results are given as the mean (SD), and 95% confidence interval (CI; confidence interval, U; upper, L; lower).

Parameter		RapidNP	RapidP	Slow
VCL (µm/s)	Mean	154.1 ^a^	173.0 ^b^	28.6 ^c^
	SD	77.1	61.1	10.1
	CI-L	149.5	171.8	28.1
	CI-U	158.6	174.3	29.1
VSL (µm/s)	Mean	41.6 ^a^	119.1 ^b^	5.41 ^c^
	SD	31.1	38.7	7.78
	CI-L	39.8	118.3	5.03
	CI-U	43.4	119.8	5.80
VAP (µm/s)	Mean	83.0 ^a^	126.7 ^b^	10.7 ^c^
	SD	49.7	38.9	8.53
	CI-L	80.1	125.9	10.3
	CI-U	85.9	127.5	11.1
LIN (%)	Mean	24.8 ^a^	70.8 ^b^	18.0 ^c^
	SD	14.4	13.9	21.6
	CI-L	24.0	70.5	17.0
	CI-U	25.7	71.1	19.1
STR (%)	Mean	45.6 ^a^	93.5 ^b^	42.6 ^c^
	SD	19.4	6.16	33.6
	CI-L	44.5	93.3	40.9
	CI-U	46.7	93.6	44.3
WOB (%)	Mean	50.9 ^a^	75.4 ^b^	36.1 ^c^
	SD	18.1	12.6	23.2
	CI-L	49.9	75.1	35.1
	CI-U	52.0	75.6	37.4
ALH (µm)	Mean	4.22 ^a^	3.22 ^b^	1.28 ^c^
	SD	1.74	1.40	0.43
	CI-L	4.12	3.19	1.26
	CI-U	4.32	3.25	1.31
BCF (Hz)	Mean	20.5 ^a^	29.2 ^b^	7.81 ^c^
	SD	7.57	7.35	6.70
	CI-L	20.0	29.1	7.47
	CI-U	20.9	29.4	8.14

^a–c^ Values in rows with distinct letters differ significantly (*p* < 0.05).

## Data Availability

Data are contained within the article. Raw data are available on request by the author.

## Supplementary Materials

The following supporting information can be downloaded at: https://www.mdpi.com/article/10.3390/biology12081086/s1, Figure S1: Distribution of sperm cells within subpopulations: immotile, rapid non-progressive (RapidNP), rapid progressive (RapidP), and Slow motile, for fresh and frozen–thawed (FT) semen from different bulls (*n* = 9). The results are shown as the mean percentage motile sperm cells of the different populations. Figure S2: Distribution of the percentage of hyperactive (Hyp) motile sperm cells from fresh semen of nine NRF bulls analyzed after incubation at 38 °C for 5 min (T0), three hours (T3) and six hours (T6) in spTalp-H. The results are presented as the mean (±95% confidence interval).
