# Supplementary material for: Motility Subpopulations with Distinct Motility Characteristics Using Swim-Up-Selected Sperm Cells from Norwegian Red Bulls: Effects of Freezing–Thawing and Between-Bull Variation
Source: Biology (Basel). 2023 Aug 3;12(8):1086. doi: 10.3390/biology12081086 (PMC10452253; doi:10.3390/biology12081086)
Supplement: Supplementary file 1 [file biology-12-01086-s001.zip › biology-2488759-FigureS2.pdf]

Supplementary Figure S2

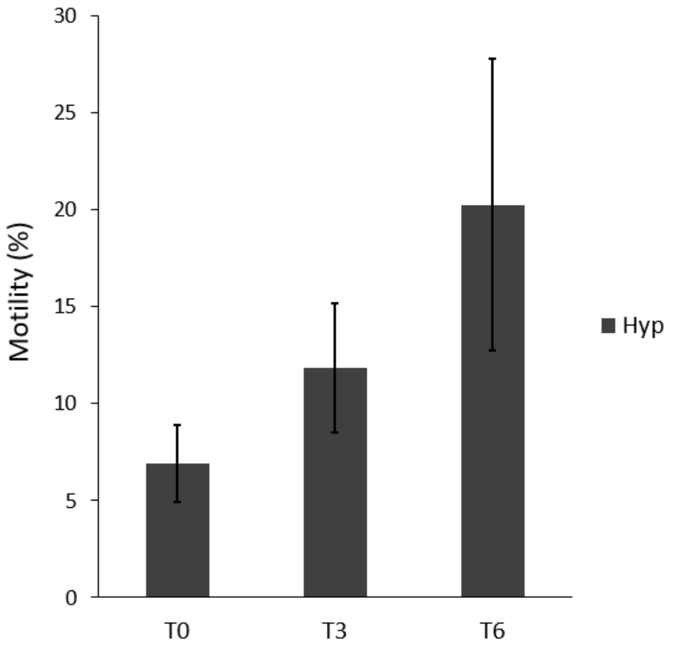

Figure S2. Distribution of percentage hyperactive (Hyp) motile sperm cells from fresh semen from nine NRF bulls analyzed after incubation at 38°C for 5 min (T0), three hours (T3) and six hours (T6) in spTalp-H. Results are presented as mean (±95% confidence interval).
