# Supplementary figures and images for: Motility Subpopulations with Distinct Motility Characteristics Using Swim-Up-Selected Sperm Cells from Norwegian Red Bulls: Effects of Freezing–Thawing and Between-Bull Variation
Source: Biology (Basel). 2023 Aug 3;12(8):1086. doi: 10.3390/biology12081086 (PMC10452253; doi:10.3390/biology12081086)

Supplementary figure 1a

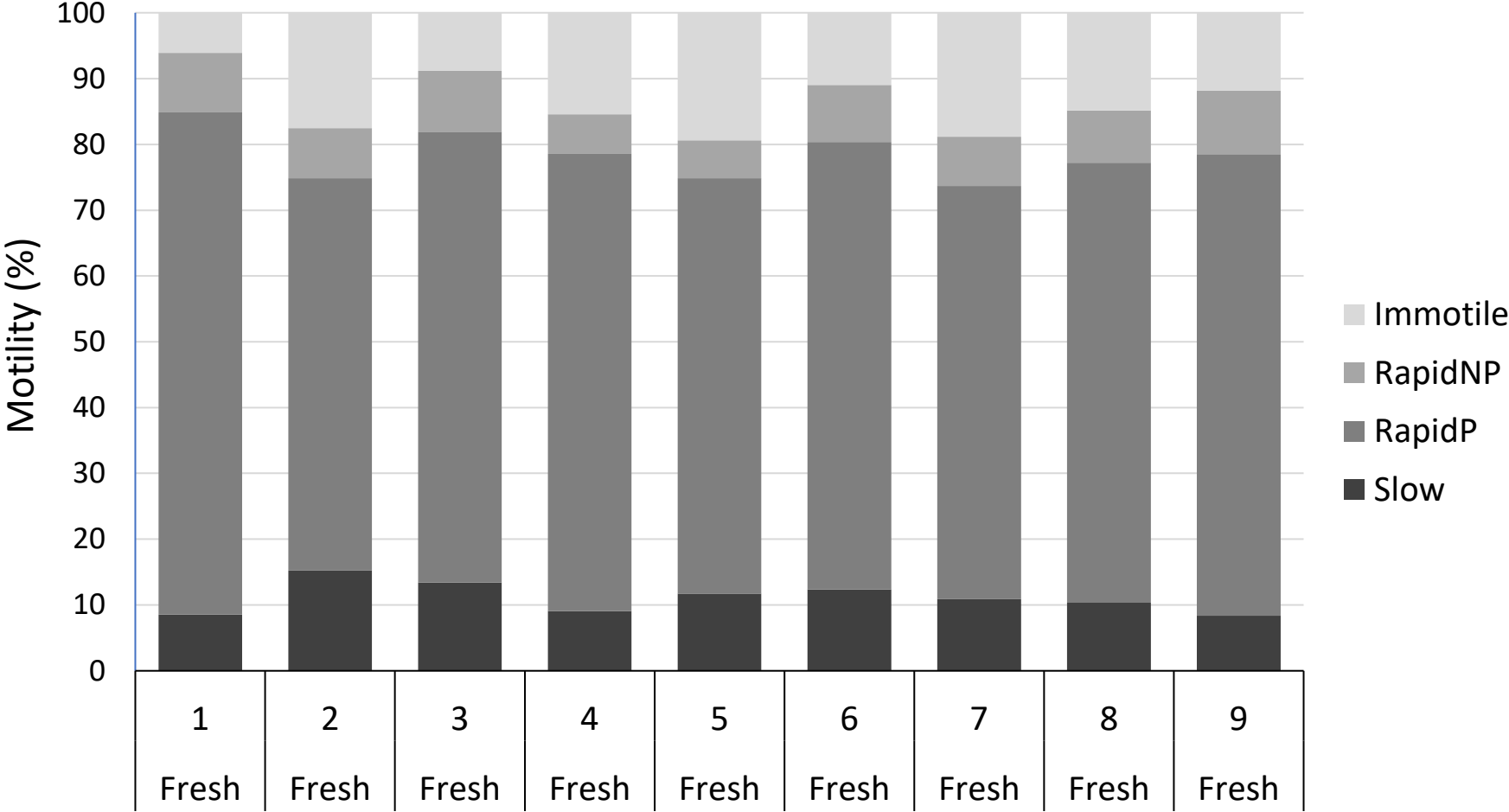

(a)

Supplementary figure 1b

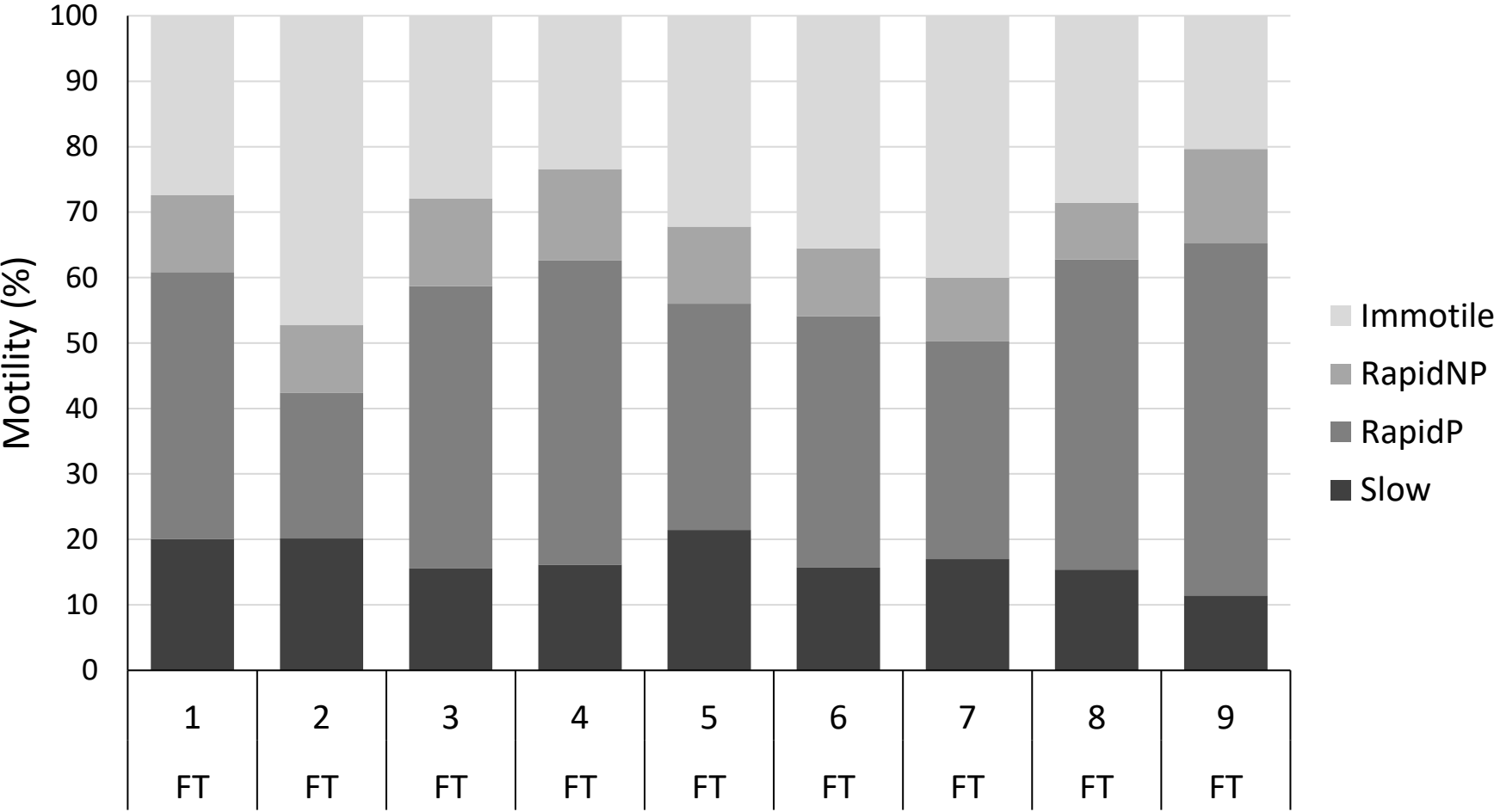

(b)

Supplement: Supplementary file 1 [file biology-12-01086-s001.zip › biology-2488759-FigureS1.pdf]
